# The Endothelin Receptor Antagonist Macitentan Improves Isosorbide-5-Mononitrate (ISMN) and Isosorbide Dinitrate (ISDN) Induced Endothelial Dysfunction, Oxidative Stress, and Vascular Inflammation

**DOI:** 10.1155/2018/7845629

**Published:** 2018-12-27

**Authors:** Sebastian Steven, Matthias Oelze, Michael Hausding, Siyer Roohani, Fatemeh Kashani, Swenja Kröller-Schön, Johanna Helmstädter, Thomas Jansen, Christine Baum, Marc Iglarz, Eberhard Schulz, Thomas Münzel, Andreas Daiber

**Affiliations:** ^1^Center for Cardiology, Department of Cardiology, University Medical Center Mainz, Langenbeckstr. 1, 55131 Mainz, Germany; ^2^Center of Thrombosis and Hemostasis, University Medical Center Mainz, Langenbeckstr. 1, 55131 Mainz, Germany; ^3^Actelion Pharmaceuticals Deutschland GmbH, Konrad-Goldmann-Straße 5b, 79100 Freiburg, Germany; ^4^Pharmacology and Preclinical Development, Actelion Pharmaceuticals Ltd., Gewerbestrasse 16, CH-4123 Allschwil, Switzerland; ^5^Partner Site Rhine-Main, German Center for Cardiovascular Research (DZHK), Langenbeckstr. 1, 55131 Mainz, Germany

## Abstract

**Objective:**

Organic nitrates such as isosorbide-5-mononitrate (ISMN) and isosorbide dinitrate (ISDN) are used for the treatment of patients with chronic symptomatic stable coronary artery disease and chronic congestive heart failure. Limiting side effects of these nitrovasodilators include nitrate tolerance and/or endothelial dysfunction mediated by oxidative stress. Here, we tested the therapeutic effects of the dual endothelin (ET) receptor antagonist macitentan in ISMN- and ISDN-treated animals.

**Methods and Results:**

Organic nitrates (ISMN, ISDN, and nitroglycerin (GTN)) augmented the oxidative burst and interleukin-6 release in cultured macrophages, whereas macitentan decreased the oxidative burst in isolated human leukocytes. Male C57BL/6j mice were treated with ISMN (75 mg/kg/d) or ISDN (25 mg/kg/d) via s.c. infusion for 7 days and some mice in addition with 30 mg/kg/d of macitentan (gavage, once daily). ISMN and ISDN *in vivo* therapy caused endothelial dysfunction but no nitrate (or cross-)tolerance to the organic nitrates, respectively. ISMN/ISDN increased blood nitrosative stress, vascular/cardiac oxidative stress via NOX-2 (fluorescence and chemiluminescence methods), ET1 expression, ET receptor signaling, and markers of inflammation (protein and mRNA level). ET receptor signaling blockade by macitentan normalized endothelial function, vascular/cardiac oxidative stress, and inflammatory phenotype in both nitrate therapy groups.

**Conclusion:**

ISMN/ISDN treatment caused activation of the NOX-2/ET receptor signaling axis leading to increased vascular oxidative stress and inflammation as well as endothelial dysfunction. Our study demonstrates for the first time that blockade of ET receptor signaling by the dual endothelin receptor blocker macitentan improves adverse side effects of the organic nitrates ISMN and ISDN.

## 1. Introduction

Organic nitrates such as glyceryl trinitrate (GTN), isosorbide dinitrate (ISDN), and isosorbide-5-mononitrate (ISMN) still remain the foremost orally available drugs in the treatment of patients with chronic symptomatic stable coronary artery disease, acute myocardial infarction, and chronic congestive heart failure [1–3]. The most limiting side effects include the development of nitrate tolerance and/or endothelial dysfunction, which has been described to occur in response to chronic treatment of humans with GTN, ISDN, and ISMN [2, 4–6]. While the pathophysiology underlying GTN-induced endothelial dysfunction has been extensively characterized [1, 2], especially regarding the adverse effects on the hemodynamic and autonomic response [7, 8], there are only limited data available to explain why ISMN or ISDN has adverse effects on the vascular endothelium. In contrast to GTN and pentaerithrityl tetranitrate (PETN), ISMN and ISDN are not subject to bioactivation by the mitochondrial aldehyde dehydrogenase (ALDH-2) located within mitochondria [9, 10]. Other ALDH isoforms were identified to bioactivate various organic nitrates in vitro [11, 12], and it is postulated that cytosolic ALDH-2 is responsible at least in some cell types (e.g., vascular cells) for GTN bioactivation [13]. ISDN and GTN are supposed to be bioactivated by P450 enzymes [14, 15]. In addition, xanthine oxidoreductase was demonstrated as an ISDN- and ISMN-metabolizing enzyme with higher turnover with xanthine instead of NADH as the source of electrons, although only at suprapharmacological organic nitrate levels [16].

ISMN treatment causes endothelial dysfunction in human subjects, which is corrected completely by vitamin C administration suggesting a crucial role of reactive oxygen species (ROS) in mediating this phenomenon [17]. Recently, we have demonstrated that ISMN therapy causes endothelin-1 (ET-1) upregulation, NOX-2 activation, and eNOS uncoupling [18]. Very similar side effects, including augmented vascular ET-1 levels, were also reported for GTN therapy in animals [19, 20] and association of ET-1 mRNA with ISDN-induced tolerance in humans [21]. In contrast, other groups failed to observe upregulation of ET-1 in response to ISMN in vivo administration to rabbits [22] and were not able to prevent GTN-induced in vitro tolerance via the ET_A_ receptor antagonist [23] and did not observe supersensitivity to the vasoconstrictor ET-1 in human internal mammary artery from patients with nitrate therapy [24] or in isolated bovine coronary arteries upon induction of GTN in vitro tolerance [25]. For the nitroxyl anion donor Angeli's salt, it was reported that it may overcome ET-1-induced vascular dysfunction in murine aorta, whereas GTN failed to show beneficial effects [26]. Previous findings on nitrate tolerance (mainly in response to GTN) with respect to activation of the endothelin-1 system and its interaction with oxidative stress are also summarized in several review articles [1, 2, 27]. Observations by others on GTN-induced activation of ROS formation in whole blood pointed towards a role of nitrates in white blood cell activation [28]. With our recent studies, we could demonstrate that phagocytic NADPH oxidase in circulating white blood cells is increased under chronic GTN and ISMN therapy [18, 29] and that the extent of ISMN-induced vascular complications is synergistically increased in the setting of type 1 diabetes mellitus and arterial hypertension [30, 31]. Others at least observed no beneficial effects of ISMN therapy on atherosclerosis and endothelial dysfunction in cholesterol-fed rabbits [32]. Almost nothing is known about the mechanism of ISDN-induced side effects so far, and to our knowledge, no preclinical study has ever demonstrated that ISDN may cause endothelial dysfunction and whether this may be also linked to increased oxidative stress and increased endothelin expression.

The simultaneous upregulation of the vasoconstrictor ET-1 and increase in vascular oxidative stress can be best explained by the fact that ET-1 increases vascular superoxide formation via activation of the NADPH oxidase and stimulates inflammatory processes in mild hypertension models [33, 34]. Vice versa, it was also shown that NADPH oxidase-derived superoxide formation potentiates the vasoconstrictor properties of ET-1 [35] and that oxidative stress induces the ET-1 promoter and hence its expression [36, 37]. This provides the basis for a crosstalk or vicious circle for vasoconstrictor and oxidative stress pathways under organic nitrate therapy (reviewed in [2, 38]). In our own studies, we observed activation of leukocytes in response to ISMN in vivo therapy as well as in vitro challenges with ISMN, exogenous ET-1, or the ET_A_ receptor agonist BQ-3020 [18, 39], which were blocked by bosentan cotherapy in selected experiments.

Chronic ISMN and ISDN therapy of mice is a suitable model to test the hypothesis of this crosstalk involving ET-1 and oxidative stress/inflammation in more detail. With the present study, we further elucidated the underlying mechanisms of the effects of ISMN and ISDN therapy on ET-1 signaling, NOX-2 activation, inflammation, and vascular dysfunction by cotreatment with the endothelin receptor antagonist macitentan, displaying slow apparent receptor association kinetics resulting in a significantly lower receptor dissociation rate and longer receptor occupancy half-life [40].

## 2. Materials and Methods

### 2.1. Reagents

Isosorbide dinitrate (ISDN; 50% (*w*/*w*) with 50% (*w*/*w*) lactose) was of analytical grade and obtained from Sigma-Aldrich or Fluka. Isosorbide-5-mononitrate (ISMN) was from LKT Laboratories (St. Paul, MN, USA). Macitentan (ACT-064992; N-[5-(4-bromophenyl)-6-(2-(5-bromopyrimidin-2-yloxy)ethoxy)pyrimidin-4-yl]-N′-propylaminosulfamide) was a kind gift of Actelion Pharmaceuticals Ltd. (Allschwil, Switzerland). For isometric tension studies, GTN was obtained from a Nitrolingual infusion solution (1 mg/ml) from G. Pohl-Boskamp (Hohenlockstedt, Germany). The Bradford reagent was obtained from Bio-Rad (Munich, Germany). All other chemicals were obtained from Fluka, Sigma-Aldrich, or Merck.

### 2.2. Cell Culture

RAW 264.7 cell macrophages were purchased from LGC Standards (Wesel, Germany). The cells were cultured in DMEM-NM (#21885-025) containing GlutaMAX from Life Technologies GmbH/Gibco (Darmstadt, Germany) with 10% fetal calf serum (FCS, PAA), penicillin (50 IU/ml), streptomycin (50 *μ*g/ml), and 10% CO_2_ as described [41]. Upon reaching confluence, the cells were split 1 : 3. For the final experiments, the cells were seeded into 96- or 6-well plates (final number of cells per well was 1 × 10^5^) and similar numbers of wells were incubated with medium alone (basal) or increasing concentrations of organic nitrates (GTN, ISMN, and ISDN) for 24 hours. The 96-well plates were used for ROS measurement by L-012-enhanced chemiluminescence analysis (100 *μ*M) in PBS containing calcium and magnesium (1 mM). The 6-well plates were washed twice with PBS buffer, dried, and frozen in liquid nitrogen and stored at −80°C until dot blot analysis for the IL-6 content. A rabbit polyclonal antibody against IL-6 (1 : 5000, Abcam, Cambridge, UK) was used along with a secondary peroxidase-conjugated antibody against rabbit (1 : 10,000, Vector Lab., Burlingame, CA) as described [42]. Densitometric quantification of antibody-specific dots was performed with a ChemiLux Imager (CsX-1400 M, Intas, Göttingen, Germany) and the Gel-Pro Analyzer software (Media Cybernetics, Bethesda, MD).

### 2.3. Isolation of Leukocytes from Human Blood and Measurement of Oxidative Burst and Inflammation

Handling of all human material was in accordance with the Declaration of Helsinki and was approved by the local institutional Ethics Committee. Human whole blood from healthy volunteers was freshly collected in heparin-containing Monovettes (3 × 7.5 ml). Polymorphonuclear leucocytes (neutrophils, PMNs) were isolated by sedimentation of red blood cells with dextran and subsequent centrifugation on Ficoll as described previously [43]. Upon repeated hypotonic lysis of the cell pellet with pure water to eliminate the residual erythrocytes, total blood cell count and the purity of the PMN fraction were evaluated using an automated approach with a hematology analyzer KX-21N (Sysmex Europe GmbH, Norderstedt, Germany). The typical constitution of the blood cell fractions obtained by this method was previously reported by us [44]. The activation of PMN (10^4^ cells/ml) was quantified by ROS formation during oxidative burst in response to a phorbol ester derivative (PDBu, 1 *μ*M) using L-012-enhanced chemiluminescence (ECL) (100 *μ*M) in PBS with 1 mM calcium/magnesium at 37°C on a Centro plate reader, using a 96-well plate, 200 *μ*l sample per well, and the ECL signal (counts/s) at 20 min (Berthold Technology, Bad Wildbad, Germany).

Superoxide formation in stimulated PMN (10^6^ cells/ml, 10 *μ*M PDBu in PBS with 1 mM calcium/magnesium) was determined by incubation with 50 *μ*M dihydroethidium (DHE) for 30 min at 37°C and quantification of the superoxide-specific oxidation product 2-hydroxyethidium (2-HE) by high-performance liquid chromatography (HPLC) as described [45]. 50 *μ*l of the supernatant was subjected to HPLC analysis. The system consisted of a control unit, two pumps, mixer, detectors, column oven, degasser, and an autosampler (AS-2057 plus) from Jasco (Groß-Umstadt, Germany) and a C_18_-Nucleosil 100-3 (125 × 4) column from Macherey-Nagel (Düren, Germany). A high-pressure gradient was employed with 50 mM citrate buffer pH 2.2 (solvent A) and acetonitrile with 10% water (solvent B) as mobile phases with the following percentages of the organic solvent: 0 min, 40% B; 7 min, 45% B; 8–12 min, 100% B; 13 min, 40%. The flow was 1 ml/min, and DHE was detected by its absorption at 355 nm, whereas 2-hydroxyethidium and ethidium were detected by fluorescence (Ex. 480 nm/Em. 580 nm). The effect of macitentan on ROS and superoxide formation was determined by coincubation with increasing macitentan concentrations (1-1000 *μ*M).

### 2.4. Animals and In Vivo Treatment

All animals were treated in accordance with the *Guide for the Care and Use of Laboratory Animals* as adopted by the US National Institutes of Health. All animal experimental protocols were reviewed by the Ethics Committee of the Johannes Gutenberg University Medical Center and approved by the Landesuntersuchungsamt (Koblenz, Germany; #23177-07/G 12-1-084 and G 14-1-028). All tissue preparations were performed according to established standards for the ethical treatment of animals. Male Wistar rats (8 weeks old, 350 g) were obtained from Charles River Laboratories (Sulzfeld, Germany) and were housed according to standard operating procedures in the animal facility of the Johannes Gutenberg University and were treated with ISDN (0, 10, 25, or 50 mg/kg/d) via administration in the drinking water for 7 days. Male C57BL/6j mice (10-12 weeks old, 25-30 g) were obtained from our own in-house animal facility and were housed according to standard operating procedures in the animal facility of the Johannes Gutenberg University. Mice were treated with ISMN (75 mg/kg/d) or ISDN (25 mg/kg/d) or the vehicle alone (dimethyl sulfoxide (DMSO)) via subcutaneous osmotic mini pumps (model 2007, ALZET, Cupertino, USA) for 7 days [31, 45]. All surgery was performed under ketamine/xylazine anesthesia (1 ml/kg of a ready to use solution containing 80 mg/ml ketamine and 12 mg/ml xylazine, Sigma-Aldrich). Control pumps were applied with the solvent (DMSO). For cotherapy, we used an oral dose of 30 mg/kg/d for the macitentan therapy (gavage, once daily) [46]. After one week of organic nitrate treatment with or without macitentan treatment, rats or mice were killed by exsanguination under isoflurane anesthesia, and the blood, aorta, and heart were collected.

### 2.5. Isometric Tension Recordings

The method was first described in [47]. Perivascular fat was removed from every thoracic aorta, and two ring segments (length 5-6 mm) were used for isometric tension studies of each mouse or rat. The ring segments were preconstricted by KCl in a concentration-dependent fashion (80 mM maximal concentration) in order to test the functional integrity of the tissue. Next, the ring segments were preconstricted by prostaglandin F_2*α*_ (2-4 *μ*M) for mice or phenylephrine (0.5-1 *μ*M) for rats yielding a stable plateau constriction of 50-60% of the maximal KCl-dependent vasoconstriction. Concentration-relaxation curves in response to increasing concentrations of acetylcholine (ACh), ISMN, ISDN, or GTN were performed as described [18, 48, 49]. Concentration-constriction curves in response to increasing concentrations of ET-1 were performed with aortic rings without preconstriction as described [18].

### 2.6. Detection of Nitrosative Stress in Whole Blood by EPR Spectroscopy

Organic nitrate-derived ^·^NO levels in whole blood were measured using electron paramagnetic resonance (EPR) spectroscopy by nitrosyl-iron hemoglobin (HbNO) [50]. HbNO can be regarded as a read-out of nitrosative stress in whole blood as previously reported for GTN-induced tolerance in rats [51] or lipopolysaccharide-triggered endotoxemia in rats and mice [52]. Samples of venous blood were obtained by cardiac puncture of anesthetized rats; blood samples were snap frozen and later on stored in liquid nitrogen. The EPR measurements were carried out at 77 K using an X-band table-top spectrometer MS400 (Magnettech, Berlin, Germany). The instrument settings were as follows: 10 mW microwave power, 7000 mG amplitude modulation, 100 kHz modulation frequency, 3300 G center field, 300 G sweep width, 60 s sweep time, and 3 scans. HbNO levels were expressed as arbitrary units of intensity of the first peak of the characteristic triplet signal.

### 2.7. Dot Blot and Western Blot Analysis

The levels of 3-nitrotyrosine- (3-NT-) positive proteins and F4/80 expression were assessed by dot blot analysis [31, 53]. For detection of 3-NT, a primary mouse monoclonal nitrotyrosine antibody (Millipore, Billerica, USA) was used at a dilution of 1 : 1000. For imaging of F4/80, a primary rat monoclonal F4/80 antibody (eBioscience, San Diego, CA, USA) was used at a dilution of 1 : 250. Detection and quantification were performed by ECL with peroxidase-conjugated secondary antibodies against mouse or rat (1 : 10,000, Vector Lab., Burlingame, CA). Densitometric quantification of antibody-specific dots was performed as described above.

Isolated aortic tissue from mice was frozen in liquid nitrogen and homogenized in buffer (Tris-HCl 20 mM, saccharose 250 mM, ethylene glycol-bis (*β*-aminoethyl ether)-N,N,N′,N′-tetraacetic acid (EGTA) 3 mM, ethylene diamine tetraacetic acid (EDTA) 20 mM, protease inhibitor cocktail (Roche complete, 1 tablet in 100 ml), and Triton-X-100 1 *v*/*v*%). Proteins were separated by SDS-PAGE and blotted onto nitrocellulose membranes [18, 53]. After blocking, immunoblotting was performed with the following antibodies: polyclonal mouse *β*-actin (42 kDa) and *α*-actinin (100 kDa) as a control for loading and transfer (both from Sigma-Aldrich, USA), monoclonal mouse NADPH oxidase 2 (NOX-2, 1 : 500, BD Bioscience, USA), and polyclonal rabbit ET_B_ receptor (ET_B_, 1 : 5000, Abcam, USA). Detection and quantification were performed by ECL with peroxidase-conjugated secondary antibodies against mouse or rabbit (1 : 10,000, Vector Lab., Burlingame, CA). Densitometric quantification was performed as described above.

### 2.8. Immunohistochemical (IHC) Analysis of ET-1 Expression

Aortic samples were fixed in paraformaldehyde (4%), embedded in paraffin, and stained with a primary mouse antibody against ET-1 (Pierce #MA3-005: 1 : 200). Biotinylated secondary antibody (anti-mouse included in M.O.M. Kit, Vector Lab., Burlingame) was used at a dilution according to the manufacturer's instructions. For immunochemical detection, ABC reagent (Vector) and then DAB reagent (peroxidase substrate kit, Vector) were used as substrates.

### 2.9. Assessment of Oxidative Stress and NADPH Oxidase Activity in the Heart and Aorta

NADPH oxidase activity in membrane fractions of heart tissue was determined by lucigenin (5 *μ*M) ECL in the presence of 200 *μ*M NADPH [42, 43]. For ROS formation in aortic tissue, isolated aortic ring segments were OCT-embedded (Tissue-Tek, USA), and upon staining with dihydroethidium (DHE, 1 *μ*M), oxidative fluorescence microtopography was determined as reported [53, 54].

### 2.10. Reverse Transcription Real-Time PCR (qRT-PCR)

mRNA expression was analyzed with quantitative real-time RT-PCR as previously described [41, 42, 54]. Briefly, total RNA from the mouse aorta or heart was isolated (RNeasy Fibrous Tissue Mini Kit; Qiagen, Hilden, Germany), and 50 ng of total RNA was used for real-time RT-PCR analysis with the QuantiTectTM Probe RT-PCR kit (Qiagen). TaqMan® Gene Expression assays for NADPH oxidase isoform 2 (NOX-2), endothelin-1b receptor (ET_B_R), endothelin-converting enzyme-1 (ECE-1), monocyte chemoattractant protein-1 (MCP-1), CD11b, interleukin-6 (IL-6), and TATA box-binding protein (TBP) were purchased as probe and primer sets (Applied Biosystems, Foster City, CA). The comparative Ct method was used for relative mRNA quantification. Gene expression was normalized to the endogenous control (TBP mRNA), and the amount of target gene mRNA expression in each sample was expressed relative to that of control.

### 2.11. Statistical Analysis

Results are expressed as mean ± SEM. Two-way ANOVA (with Bonferroni's correction for comparison of multiple means) was used for comparisons of vasodilator potency and efficacy. One-way ANOVA (with Bonferroni's or Dunn's correction for comparison of multiple means) was used for comparisons of all other parameters. *p* values < 0.05 were considered as statistically significant.

## 3. Results

### 3.1. Studies with Cultured and Isolated Immune Cells

**Figure 1:**
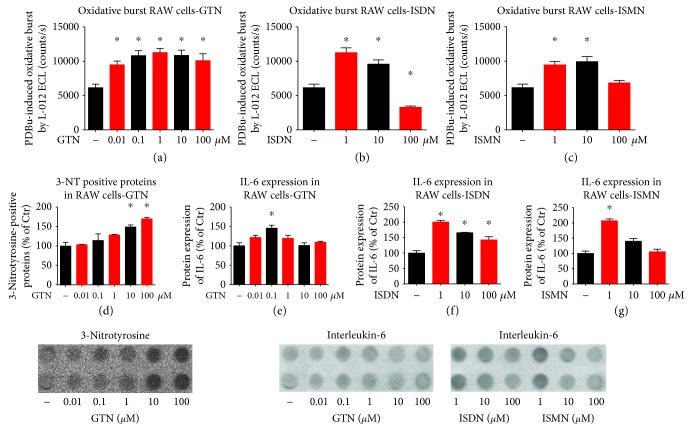
Characterization of in vitro effects of organic nitrates on oxidative burst signals and inflammatory activity of cultured RAW 264.7 cell macrophages. The organic nitrates GTN, ISDN, and ISMN increased the ROS formation by cultured macrophages in response to phorbol ester (PDBu) as measured by L-012 ECL (a–c) and 3-nitrotyrosine immunostaining (d). Likewise, GTN, ISDN, and ISMN increased the release of the cytokine IL-6 by cultured macrophages as measured by immunoblotting (e–g). The data are mean ± SEM from at least eight different cell culture wells. ^∗^*p* < 0.05 vs. solvent control.

**Figure 2:**
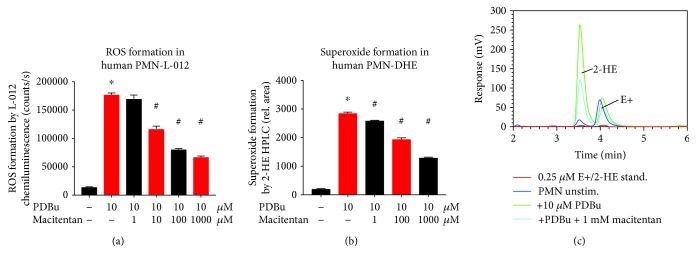
Characterization of in vitro effects of macitentan on oxidative burst signals of isolated human leukocytes. The ET receptor blocker macitentan suppressed the ROS formation by isolated human neutrophils in response to phorbol ester (PDBu) in a concentration-dependent fashion as measured by L-012 ECL (a). Likewise, macitentan suppressed the neutrophil-derived superoxide formation as measured by HPLC-based quantification of the superoxide-specific product 2-hydroxyethidium (b). Representative chromatograms are shown for selected experiments (c). The data are mean ± SEM from 8 (a) and 3 (b) independent experiments. ^∗^*p* < 0.05 vs. solvent control; ^#^*p* < 0.05 vs. PDBu-stimulated group.

In cultured RAW 264.7 cell macrophages, GTN, ISDN, and ISMN increased the oxidative burst signal in these macrophages upon phorbol ester stimulation (Figures 1(a)–1(c)). Suprapharmacological concentrations of ISDN and ISMN suppressed the ROS signal (Figures 1(b) and 1(c)), likely due to the release of ^·^NO quenching the superoxide anions. Accordingly, we established higher protein tyrosine nitration levels at higher concentrations of GTN (Figure 1(d)). All organic nitrates increased the IL-6 expression in these macrophages, at least at one specific concentration (Figures 1(e)–1(g)). The endothelin receptor blocker macitentan suppressed the superoxide signal in phorbol ester-stimulated human granulocytes (PMN) (Figure 2), indicating a role of endothelin signaling in this process.

### 3.2. Pilot Study for ISDN in Rats

**Figure 3:**
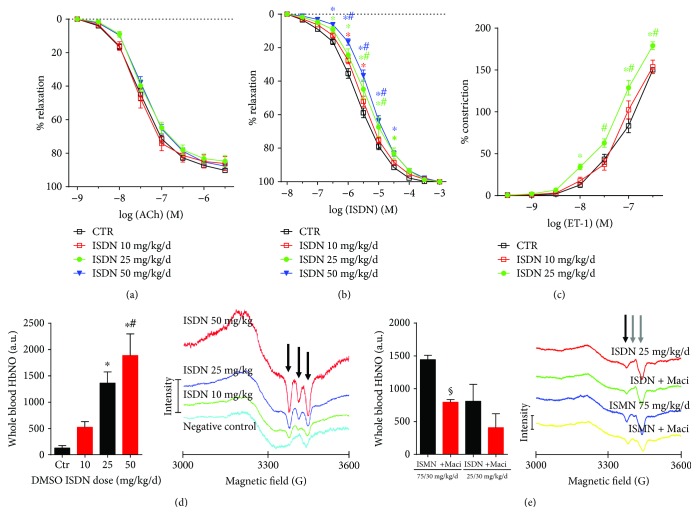
Pilot studies on the effective ISDN dose and administration protocol. Effects of 3 ISDN doses (10, 25, and 50 mg/kg/d) on endothelial function of rat aortic ring segments were tested by endothelium-dependent relaxation (ACh, a). Impact of the ISDN dose on endothelium-independent relaxation (ISDN) was determined by isometric tension studies in rat aortic ring segments in order to measure nitrate tolerance (b). Sensitivity to ET-1-dependent vasoconstriction was measured by increases in tone of rat aortic ring segments in response to cumulative concentrations of ET-1 (c). ISDN dose-dependent increase in the nitrosative stress marker nitrosyl-iron hemoglobin (HbNO) in whole blood of treated rats was measured by EPR spectroscopy quantification of the characteristic triplet signal (see representative spectra) (d). ISMN (75 mg/kg/d) and ISDN (25 mg/kg/d) treatment increases HbNO in whole blood of treated mice, which is decreased by macitentan therapy (quantification of the left peak of the triplet signal, see representative spectra) (e). The data are mean ± SEM from 9-12 (a, b), 6-8 (c), 5-6 (d), and 3-4 (e) independent experiments. ^∗^*p* < 0.05 vs. solvent control; ^#^*p* < 0.05 vs. 10 mg/kg/d ISDN group; ^§^*p* < 0.05 vs. 75 mg/kg/d ISMN group.

Since we had no previous expertise on ISDN in vivo treatment of animals, we performed a dose-response study in rats. ISDN was administrated at increasing doses in the drinking water. The ISDN dose of 25 and 50 mg/kg/d induced no significant endothelial dysfunction, although a trend of impaired acetylcholine-dependent relaxation was visible (Figure 3(a)). In contrast, the ISDN-dependent relaxation was significantly impaired in response to 25 and 50 mg/kg/d of ISDN (and the 10 mg/kg/d in one point of the concentration-relaxation curve) indicating a mild nitrate tolerance for the higher doses of ISDN (Figure 3(b)). Treatment with ISDN at 25 mg/kg/d also caused supersensitivity to the vasoconstrictor ET-1 (Figure 3(c)). Successful uptake of the drug in an effective dose was tested by measurement of nitrosyl-iron hemoglobin (HbNO), a marker of whole blood nitrosative stress due to high levels of NO-derived nitrosating species as previously reported by us in endotoxin-induced sepsis or GTN-induced nitrate tolerance. ISDN treatment dose dependently increased the HbNO levels in whole blood of rats proving the successful delivery of the drug at all doses (Figure 3(d)). Based on our previous experience on successful s.c. administration of ISMN to mice via osmotic mini pumps and the present findings on lack of endothelial dysfunction in orally ISDN-treated rats, we decided to use the s.c. protocol for ISMN and ISDN administration in our mouse model. Successful uptake of ISMN (75 mg/kg/d) and ISDN (25 mg/kg/d) in effective doses was tested by measurement of nitrosyl-iron hemoglobin (HbNO). Both organic nitrates induced a clearly detectable HbNO signal, which was almost 2-fold higher in the ISMN group (Figure 3(e)). Macitentan reduced the nitrosative stress signal in both groups by approximately 50%, at least by trend.

### 3.3. ISMN In Vivo Treatment

#### 3.3.1. Vascular Function and Cardiovascular Oxidative Stress Parameters in the Mouse Aorta

**Figure 4:**
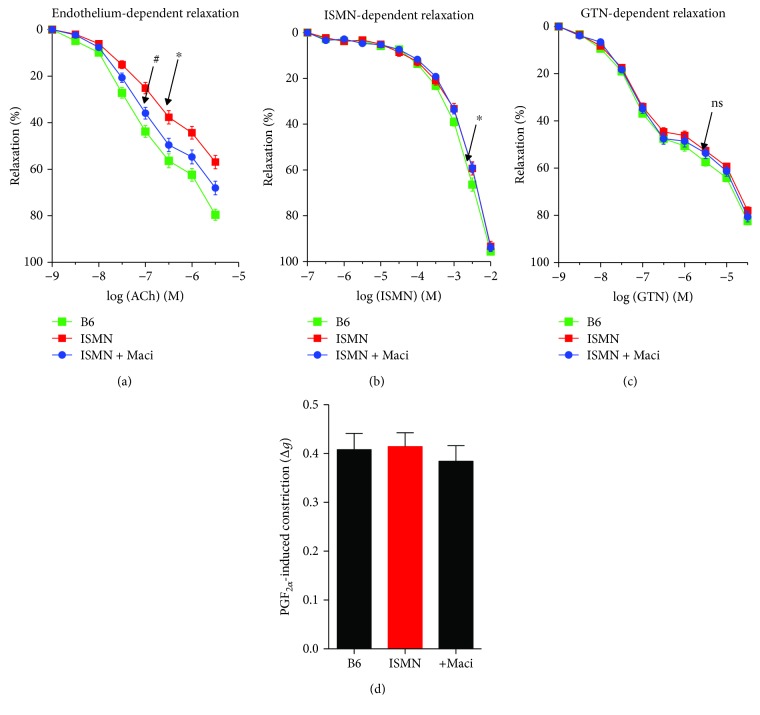
Characterization of vascular function and protective effects of macitentan cotherapy in ISMN-treated mice. Endothelium-dependent relaxation (ACh) was determined by isometric tension studies in aortic ring segments in order to assess endothelial function (a). Endothelium-independent relaxation (ISMN, GTN) was determined by isometric tension studies in aortic ring segments in order to measure nitrate tolerance and cross-tolerance (b, c). Vasoconstriction was determined by prostaglandin F_2*α*_ (PGF_2*α*_) (d). The data are mean ± SEM from aortic ring segments of 15-21 (a), 12-16 (b), 13-19 (c), and 16-24 (d) mice per group. ^∗^*p* < 0.05 vs. control; ^#^*p* < 0.05 vs. ISMN group.

**Figure 5:**
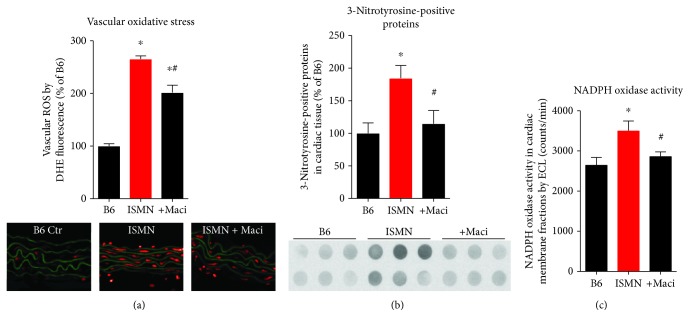
Characterization of vascular/cardiac oxidative stress and protective effects of macitentan cotherapy in ISMN-treated mice. DHE (1 *μ*M) oxidative fluorescence microtopography was used to assess vascular oxidative stress (a). Representative staining images are shown below the densitometric quantification, and the green fluorescence represents autofluorescence of the cytoplasmic membranes. Levels of 3-NT-positive proteins in cardiac tissue were assessed by dot blot analysis and specific antibodies (b). Representative blots are shown below the densitometric quantification. Cardiac NADPH oxidase (NOX) activity was measured by the chemiluminescence probe lucigenin in the presence of NADPH (c). The data are mean ± SEM from at least 6 aortas (a), 8-9 hearts (b), and 5 pooled samples of at least 15 (c) mice per group. ^∗^*p* < 0.05 vs. control; ^#^*p* < 0.05 vs. ISMN group.

ISMN treatment caused endothelial dysfunction but no appreciable tolerance to ISMN and no cross-tolerance to GTN (Figures 4(a)–4(c)). Endothelial dysfunction was improved by macitentan cotherapy (Figure 4(a)). The sensitivity of the vasculature to prostaglandin F_2*α*_-dependent constriction was neither affected by ISMN treatment nor by macitentan cotherapy (Figure 4(d)). ISMN increased aortic ROS production that was corrected by macitentan cotherapy (Figure 5(a)). Cardiac protein tyrosine nitration, as a marker for *in vivo* peroxynitrite formation, was increased by ISMN therapy and normalized by macitentan (Figure 5(b)). Cardiac NADPH oxidase activity was increased in response to ISMN and normalized by macitentan (Figure 5(c)). However, these oxidative stress parameters were somewhat more efficiently normalized in cardiac tissue as compared to aortic tissue, suggesting the contribution of different ROS sources to ISMN-mediated oxidative damage in these tissues.

#### 3.3.2. Protein and mRNA Expression

**Figure 6:**
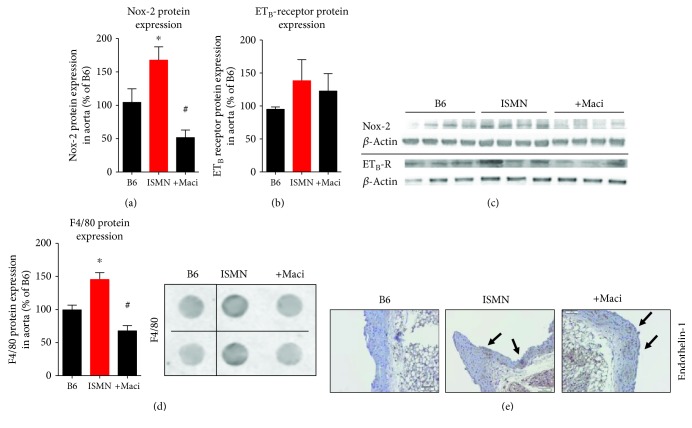
Characterization of vascular protein expression within the inflammatory and ET receptor signaling pathways and protective effects of macitentan cotherapy in ISMN-treated mice. The vascular protein expression of NOX-2 (a) and ET_B_ receptor (b) was determined by western blot analysis. Representative blots are shown besides the densitometric quantification (c). The vascular protein expression of F4/80 was determined by dot blot analysis (d). Representative dot blots are shown besides the densitometric quantification. Immunohistochemical staining for ET-1 in aortic paraffin sections (e). Arrows indicate ET-1-specific brown color. Representative images for 4 independent experiments. The data are mean ± SEM from 5-6 (a), 5-6 (b), and 4-7 (c) mice per group. ^∗^*p* < 0.05 vs. control; ^#^*p* < 0.05 vs. ISMN group.

**Figure 7:**
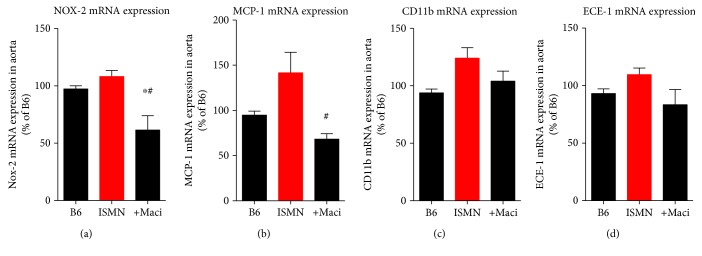
Characterization of vascular mRNA expression within the inflammatory and ET receptor signaling pathways and protective effects of macitentan cotherapy in ISMN-treated mice. qRT-PCR was used to determine mRNA expression levels of Nox-2 (a), MCP-1 (b), CD11b (c), and ECE-1 (d). The data are mean ± SEM from 9-10 (a), 8-9 (b), 6-7 (c), and 8-9 (d) mice per group. ^∗^*p* < 0.05 vs. control; ^#^*p* < 0.05 vs. ISMN group.

Aortic NOX-2 protein expression was increased by ISMN treatment and significantly decreased by macitentan (Figures 6(a) and 6(c)). The expression pattern was almost similar at the mRNA level (Figure 7(a)). The protein expression of the ET_B_ receptor was not modified (Figure 6(b)–6(c)). Inflammatory markers were increased at the protein level in the aorta of ISMN-treated mice as envisaged by higher F4/80 expression (Figure 6(d)) but also at the mRNA expression levels of MCP-1 and CD11b (Figures 7(b)–7(c)), all of which were mostly normalized by macitentan cotherapy. ET-1 protein expression was envisaged by immunohistochemistry and was more pronounced in the ISMN groups (Figure 6(e)). The mRNA expression of the endothelin-converting enzyme-1 (ECE-1) showed no significant changes (Figure 7(d)).

### 3.4. ISDN In Vivo Treatment

#### 3.4.1. Vascular Function and Cardiovascular Oxidative Stress Parameters in the Mouse Aorta

**Figure 8:**
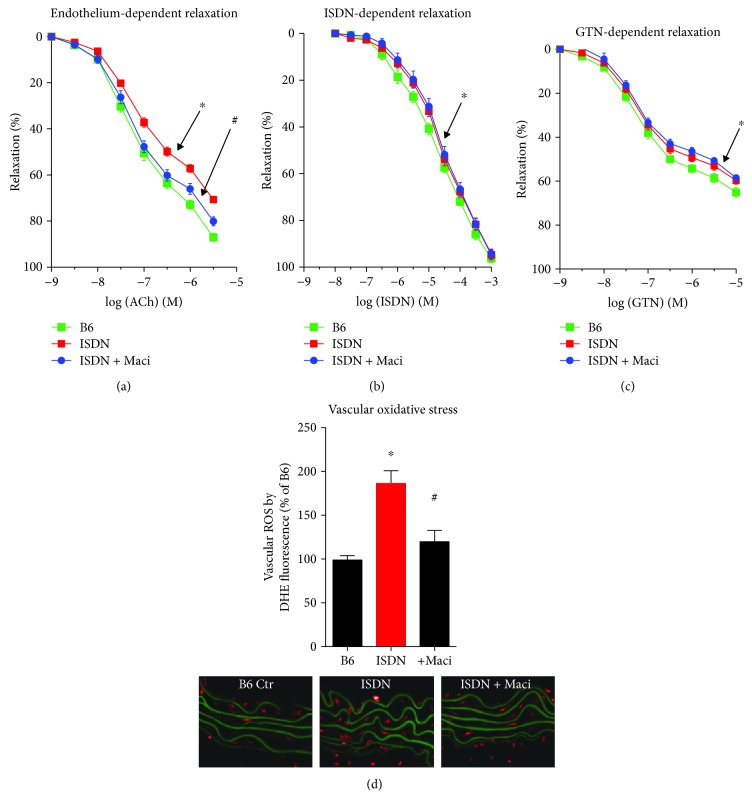
Characterization of vascular function and oxidative stress and protective effects of macitentan cotherapy in ISDN-treated mice. Endothelium-dependent relaxation (ACh) was determined by isometric tension studies in aortic ring segments in order to assess endothelial function (a). Endothelium-independent relaxation (ISDN, GTN) was determined by isometric tension studies in aortic ring segments in order to measure nitrate tolerance and cross-tolerance (b, c). DHE (1 *μ*M) oxidative fluorescence microtopography was used to assess vascular oxidative stress (d). Representative staining images are shown below the densitometric quantification, and the green fluorescence represents autofluorescence of the cytoplasmic membranes. The data are mean ± SEM from aortic ring segments of 10-12 (a, b, c) and 12 (d) mice per group. ^∗^*p* < 0.05 vs. control; ^#^*p* < 0.05 vs. ISDN group.

ISDN treatment caused endothelial dysfunction but no pronounced tolerance to ISDN and cross-tolerance to GTN (Figures 8(a)–8(c)). Endothelial dysfunction was significantly improved by macitentan cotherapy (Figure 8(a)). The prostaglandin F_2*α*_-dependent constriction was neither affected by ISDN treatment nor by macitentan cotherapy (not shown). Aortic ROS formation was significantly augmented in response to ISDN and normalized by macitentan (Figure 8(d)).

#### 3.4.2. mRNA Expression

**Figure 9:**
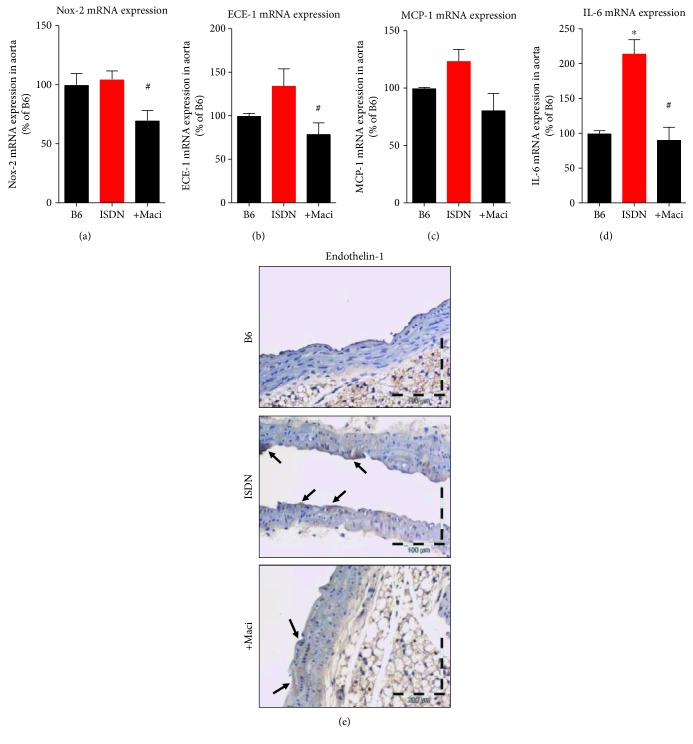
Characterization of vascular mRNA expression within the inflammatory and ET receptor signaling pathways and protective effects of macitentan cotherapy in ISDN-treated mice. qRT-PCR was used to determine mRNA expression levels of Nox-2 (a), ECE-1 (b), MCP-1 (c), and IL-6 (d). Immunohistochemical staining for ET-1 in aortic paraffin sections (e). Arrows indicate ET-1-specific brown color. Representative images for 4 independent experiments. The data are mean ± SEM from 5 (a), 8-9 (b), 3 (c), and 3 (d) mice per group. ^∗^*p* < 0.05 vs. control; ^#^*p* < 0.05 vs. ISDN group.

The mRNA expression of Nox2 was not increased by ISDN infusion but showed a robust decrease by macitentan cotherapy (Figure 9(a)). The mRNA expression of ECE-1 showed a trend of an increase under ISDN therapy and a complete normalization by cotherapy with macitentan (Figure 9(b)). The mRNA expression of the marker of inflammation, MCP-1, was increased by ISDN infusion and normalized by macitentan cotherapy, both by trend (Figure 9(c)). The mRNA level of the cytokine IL-6 was increased by ISDN infusion and normalized by cotherapy with macitentan (Figure 9(d)). Immunohistochemical staining for ET-1 revealed a minor increase in the ISDN group and a moderate decrease by macitentan therapy (Figure 9(e)

## 4. Discussion

The results of the present study demonstrate that the organic nitrates ISMN and ISDN cause an increase in whole blood nitrosative stress and vascular/cardiac oxidative stress, activate ET receptor signaling pathways, and induce an inflammatory phenotype of the vascular system leading to endothelial dysfunction. By using the dual ET receptor blocker macitentan, we were able to avoid these major cardiovascular side effects of the organic nitrate therapy (although oxidative stress parameters per se were somewhat more efficiently normalized in the heart as compared to the aorta pointing towards different ROS sources that are activated by ISMN treatment in these tissues), further supporting a key role of ET receptor signaling in these ISMN/ISDN-induced side effects. The novel aspects of the present studies, besides the evaluation of the protective effects of the dual ET receptor blocker macitentan in the setting of nitrate tolerance, are mainly based on the identification of increased ET-1 expression, supersensitivity to ET-1-dependent vasoconstriction, followed by adverse ET receptor signaling, increased oxidative stress, inflammation, and vascular dysfunction in ISDN-treated animals. ISDN-induced endothelial dysfunction was shown in humans before but without providing a mechanistic explanation [55].

Previous studies by our group and others demonstrated that GTN and ISMN therapy upregulates ET-1 expression in the vessels of animals [18–20]. The only organic nitrate so far that is devoid of the primary clinical side effects of nitrate therapy (e.g., oxidative stress and nitrate tolerance) is pentaerythritol tetranitrate (PETN) [1, 27]. This was demonstrated in healthy subjects [56], patients with pulmonary arterial hypertension [57], in patients with chronic stable angina [58], in women with preeclampsia [59], or in experimental models such as type 1 diabetes mellitus, arterial hypertension, and pulmonary hypertension [30, 31, 39, 60] as well as congestive heart failure [61, 62]. PETN treatment leads to an induction of the highly protective enzymes heme oxygenase-1 (HO-1), ferritin [63–66], glutathione peroxidase [60], and superoxide dismutase with recoupling of eNOS [67] leading to improved mobilization of endothelial progenitor cells [68]. These protective effects of PETN are not shared by GTN, ISMN, or ISDN and require the transcription factor Nrf-2 [30, 69] and are absent in HO-1 knockout mice [31]. Thus, PETN induces gene expression of antioxidant enzymes, a property not shared by GTN [70] and likely mediated by beneficial epigenetic changes [71].

In rats with pulmonary hypertension, we recently demonstrated that PETN interferes with ET-1 signaling (downregulation of ET-1, ECE-1, and ET_A_ and ET_B_ receptor mRNA expression), thereby improving cardiac and vascular function and reducing cardiovascular and pulmonary oxidative stress and inflammation [39]. Furthermore, PETN suppressed the oxidative burst induced by the ET receptor agonist BQ-3020 in whole blood leukocytes and normalized adhesion molecule (ICAM-1) mRNA expression in cultured endothelial cells (EA.hy926). These data further highlight a key role of ET receptor signaling for the adverse effects of organic nitrates and emphasize the potential benefits of treating patients with ISMN, ISDN, or GTN in combination with ET receptor blockers such as macitentan. In contrast, ET receptor blocker cotherapy will likely not further improve the clinical benefits of PETN therapy, since this organic nitrate is already devoid of most clinical side effects such as nitrate tolerance, endothelial dysfunction, and oxidative stress due to induction of intrinsic protective pathways [27, 72–74] and suppression of the ET receptor signaling pathway [39].

So far, it is not completely understood how ET-1 increases vascular superoxide formation. ET-1 activates NADPH oxidase and causes inflammation in hypertensive animals [33, 34] and, vice versa, NADPH oxidase-derived superoxide formation increases ET-1-mediated vasoconstriction [35], most likely mediated by oxidative stress-induced preproET-1 gene promoter activation [36, 37]. GTN therapy also activates protein kinase C (PKC) [19] and increased PKC activity induces the expression of the ET_B_ receptor [75]. PKC is also required for the activation of the NADPH oxidase isoforms NOX-1 and NOX-2. Further, ET-1 triggers PKC-dependent eNOS uncoupling and subsequent endothelial dysfunction; all of which was improved by bosentan therapy [76]. Thus, the NADPH oxidase pathway, especially the NOX-2 isoform, and ET-1 signaling are interconnected and can stimulate each other, leading to a vicious circle in the NOX-2/ET receptor signaling axis (for review, see [2, 38]). Activation of PKC by organic nitrates and ET-1 may even become more important in a broader context since PKC is a major regulator of dendritic cell activation and maturation playing a central role in adaptive immune responses by antigen processing and (cross-)activation of T cells [77].

NOX-2 in immune and vascular cells represents an important source of ROS, which play a major role in the pathogenesis of cardiovascular disease in general and nitrate-induced tolerance and endothelial dysfunction in particular. NOX-2 can be activated in a redox regulatory fashion by mitochondrial ROS formation as shown for GTN-induced nitrate tolerance and endothelial dysfunction [45] as well as angiotensin II-induced hypertension and the aging process [53, 78]. We therefore assessed NOX-2 expression and NOX activity as well as different markers of vascular/cardiac oxidative stress. Elevated superoxide levels react with eNOS-derived ^·^NO to ONOO^−^ [79], which reduces ^·^NO bioavailability and thereby contributes to endothelial dysfunction. We assessed nitrooxidative stress levels in cardiac tissue of ISMN-treated mice by 3-NT-positive protein content reflecting increased peroxynitrite (ONOO^−^) formation and ROS formation in vessels of ISMN- and ISDN-treated mice by DHE fluorescence microtopography. Both oxidative stress parameters were clearly increased in organic nitrate-treated animals and normalized by macitentan cotherapy.

Since the effect of macitentan on ISMN-dependent NOX-2 activation and expression was quite pronounced and went in parallel with its anti-inflammatory effects, we propose a key role for NOX-2 in the observed adverse vascular effects of ISMN (and probably also ISDN) therapy. However, since the inhibition of NOX-2 activity/expression by macitentan was even more pronounced than suppression of oxidative stress parameters by ET receptor blockade, we cannot exclude that also other ROS sources (e.g., mitochondria, NOX-1, or NOX-4) contribute to the overall oxidative stress condition induced by ISMN or ISDN therapy. The contribution of different ROS sources in the aorta and the heart may also explain why the effect of macitentan treatment on oxidative stress parameters in these tissues is not always comparable.

We also provide evidence for excessive ^·^NO generation by ISDN/ISMN in the blood (reflected by increased HbNO levels = nitrosative stress), which was previously also reported for GTN treatment [80]. Recently, we showed that aortic ^·^NO bioavailability can be decreased in GTN-treated rats [81] despite a substantial increase in ^·^NO-associated nitrosyl-iron hemoglobin (HbNO) in whole blood of these rats [51]. Accordingly, the observed partial normalization of HbNO levels in ISMN- and ISDN-treated mice by macitentan may be interpreted as a beneficial effect of the ET receptor blocker.

It is well known that organic nitrates activate immune cells and stimulate immune cell-dependent ROS formation. In our own previous studies, we established that GTN dose dependently increased the oxidative burst signals in whole blood [51] and that treatment with the AT1 receptor blocker telmisartan effectively suppressed this burst. [29]. In addition, in vivo treatment with GTN decreased the activity of the redox-sensitive enzyme mitochondrial aldehyde dehydrogenase (ALDH-2) in isolated white blood cells of GTN-treated human volunteers and rats [44] and increased whole blood free radicals [28], compatible with immune cell activation and altered redox status in these cells in response to therapy with the organic nitrate. ISMN in vivo therapy as well as in vitro challenges with ISMN, authentic ET-1, or the ET-1_A_ receptor agonist BQ-3020 caused leukocyte activation [18, 39], which was blocked by bosentan cotherapy in selected experiments.

ISMN, ISDN, and GTN stimulated the oxidative burst and release of cytokine IL-6 by cultured macrophages, and macitentan suppressed the oxidative burst signal in isolated human leukocytes. These data, together with the above mentioned inflammatory properties of ET-1 [33, 34], provide a direct link between ET receptor signaling, the onset of inflammation, and oxidative stress in response to organic nitrate therapy, further supporting the above postulated vicious circle in the NOX-2/ET receptor signaling axis.

An important observation of the present studies was that ISDN is causing endothelial dysfunction that is clearly linked to oxidative stress and activation of the ET-1 vasoconstrictor pathway. Interestingly, the combination of ISDN and hydralazine has been shown to have strong beneficial and long-lasting hemodynamic effects and has also been demonstrated to improve prognosis of chronic congestive heart failure patients [82, 83], likely because of the improvement of the nitroso-redox balance [84]. Since we demonstrated that hydralazine is a strong peroxynitrite quencher in GTN-induced vascular dysfunction [85, 86], it is reasonable to conclude that ISDN and hydralazine are not only working synergistically together from the hemodynamic point of view but also that the antioxidant properties of hydralazine may come into play, thus preventing ISDN-induced vascular dysfunction and proinflammatory effects by its antioxidant properties.

## 5. Conclusions

**Figure 10:**
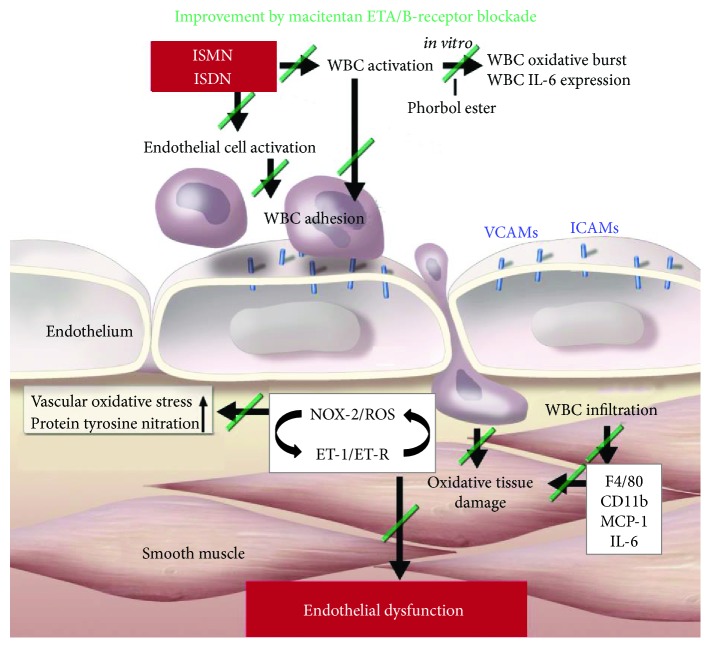
Proposed mechanism of ISMN/ISDN-induced vascular oxidative stress, inflammation, and dysfunction as well as the protective effects of macitentan. NOX-2-derived ROS formation and ET receptor signaling represent a key axis in ISMN/ISDN-induced adverse vascular effects. It is well known that ROS can induce ET-1 expression, and likewise, ET receptor signaling was shown to activate vascular NADPH oxidases and ROS formation. Thereby, the NOX-2/ET-1 signaling axis forms a vicious circle leading to further amplification of ROS formation and ET-1-mediated vascular dysfunction. Of note, ROS and ET-1 signaling are also potent triggers of vascular inflammation (for review, see [38, 89]). Accordingly, ET receptor blockade by macitentan successfully blocks white blood cell (WBC) activation and infiltration, oxidative tissue damage, and endothelial dysfunction. The scheme was modified from [90] with permission of the publisher. Copyright © 2012, Oxford University Press.

We demonstrated here for the first time that endothelial dysfunction induced by ISDN is linked to increased oxidative stress and endothelin-1 induction in vascular tissue providing a mechanistic explanation for the previously described ISDN-induced endothelial dysfunction in humans [55]. Treatment with the endothelin receptor blocker macitentan prevented endothelial dysfunction (ACh response), vascular/cardiac oxidative stress (DHE staining, 3-NT, lucigenin ECL), activation of the endothelin receptor signaling pathway, and an inflammatory phenotype of the vasculature induced by ISMN and ISDN therapy (Figure 10). Thus, our present data identified a vicious circle of the NOX-2/ET receptor signaling axis caused by ISMN/ISDN leading to augmented NOX-2 activity and ET receptor signaling. Further clinical investigations are required to demonstrate whether these side effects of ISMN/ISDN therapy may be overcome by ET receptor antagonism (e.g., by macitentan) in humans. Long-term administration of endothelin receptor antagonist atrasentan has been demonstrated to improve coronary endothelial function in patients with early atherosclerosis [87] and autocrine production of endothelin-1 accounts for 53.2% of coronary tone in advanced transplant coronary arteriosclerosis [88], making the cotherapy with endothelin receptor antagonists very attractive in patients with coronary atherosclerosis, also representing a major target group for organic nitrate therapy [1, 2]. Finally, our present data also confirm the unique properties of PETN as a clinical nitrovasodilator drug that causes induction of intrinsic antioxidant/protective pathways [27, 72, 73] and suppression of ET receptor signaling [39], emphasizing that organic nitrates are not a homogenous class of drugs [74].

## Data Availability

The datasets generated during and/or analyzed during the current study are available from the corresponding author on reasonable request.

## Abbreviations

3-NT: 3-Nitrotyrosine
ACh: Acetylcholine
DHE: Dihydroethidium
eNOS: Endothelial nitric oxide synthase
ECE-1: Endothelin-converting enzyme-1
EPR: Electron paramagnetic resonance
ET: Endothelin
ET-1: Endothelin-1
ET_A_ receptor: Endothelin receptor type A
ET_B_ receptor: Endothelin receptor type B
GTN: Nitroglycerin
HbNO: Nitrosyl-iron hemoglobin
IL-6: Interleukin-6
ISDN: Isosorbide dinitrate
ISMN: Isosorbide-5-mononitrate
MCP-1: Monocyte chemoattractant protein-1
^·^NO: Nitric oxide
NOX: NADPH oxidase
O_2_^·−^: Superoxide
PETN: Pentaerythritol tetranitrate
ROS: Reactive oxygen species
s.c.: Subcutaneously.
